# Patient experience in community health services and first choice for medical attention: A cross-sectional study in Wuhan, China

**DOI:** 10.1371/journal.pone.0288164

**Published:** 2023-07-25

**Authors:** Changmin Tang, Pengqian Fang, Xue Bai, Rui Min, Chaojie Liu

**Affiliations:** 1 School of Management, Hubei University of Chinese Medicine, Wuhan, Hubei, China; 2 Key Research Institute of Humanities and Social Sciences of Hubei Province, Wuhan, Hubei, China; 3 Tongji Medical College, Huazhong University of Science and Technology, Wuhan, Hubei, China; 4 School of Psychology and Public Health, La Trobe University, Melbourne, Australia; Public Library of Science, UNITED KINGDOM

## Abstract

**Objectives:**

In China, it is up to the patients to choose between hospitals and primary care facilities to initiate their medical care. This study aimed to determine the association between patient experience with community health centres (CHCs, a predominant provider of community-based primary care) and patient preference of taking community-based primary care facilities as a first choice for medical attention.

**Methods:**

A questionnaire survey was conducted on 1919 patients who sought medical care in 55 CHCs in Wuhan, China. Respondents were asked to identify their preferred first choice for medical attention and rate their satisfaction with eight aspects of CHCs (basic facility, medical equipment, medical services, nursing services, treatment process, courtesy and responsiveness, time spent with medical doctor, pharmacy services). Multivariate logistic regression models were established to determine the association between the CHC experience and the first choice of providers after adjustment for variations in sociodemographic characteristics.

**Results:**

Over 90% of respondents were satisfied or very satisfied with the eight aspects of CHCs; but only 75% preferred to take community-based primary care facilities as their first choice for medical attention. Those who were older and had a lower income were more likely to choose community-based primary care facilities. Geographic proximity and higher levels of satisfaction with the basic facility, courtesy and responsiveness, and pharmacy services in the CHCs were associated with a higher likelihood of taking community-based primary care facilities as a first choice for medical attention.

**Conclusion:**

The consumers of CHCs are generally satisfied with the services they received. However, one quarter of the CHC patients are yet to be convinced to accept community-based primary care facilities as a preferred first provider for medical care. Geographic proximity and patient experience with CHCs are associated with the patient choice.

## Introduction

The importance of primary health care has been recognised by researchers all over the world. Universal and equitable access to primary health care is deemed a fundamental human right [1, 2]. A strong primary health care system is critical for improving population health [3], in particular for those most vulnerable in the society [4]. The pandemic of the coronavirus disease 2019 (COVID-19) has also exposed the critical role of primary health care in the early detection of community transmissions and the control of healthcare-associated infections [5].

China has attempted to establish a primary care-led healthcare system. Community health centres (CHCs) are developed as the predominant provider of community-based primary care [6]. Over the past few decades, a large number of publicly-owned CHCs were established, including many small-sized local hospitals that were converted into CHCs. Despite the dominance of publicly-owned CHCs, private investment into primary care was also encouraged either as solo medical clinics or comprehensive CHCs [7]. Meanwhile, large hospitals were encouraged to provide community outreach services through partnerships with CHCs. Services delivered by CHCs in China are featured by their comprehensiveness as defined by the government, covering both individual-based medical care and population-oriented public health services, such as disease prevention, health education, diagnosis and treatment of common illness, rehabilitation, and management of population health (women, children, elderly, and people living with chronic conditions). Many CHCs also maintained inpatient beds.

The Chinese government envisaged community-based primary care facilities (including CHCs and independent and private clinics in the community) as a first point of contact for consumers to get access to the health care system. However, there is an absence of policy mandate to enforce such a vision and no “gatekeeping” mechanism has been put in place. Although the public are pledged to seek medical care from community-based primary care facilities, hospitals remain a more attractive choice to the public. In 2019, a total of 954,390 community-based primary care facilities were registered in China, representing an increase of 5.84% compared to the figure in 2010. Over the same period, CHCs increased by 38.50%. However, the percentage share of patient visits by primary care facilities decreased from 61.87% in 2010 to 51.96% in 2019 [8].

CHCs are expected to gain trust from community residents and enter into a non-binding voluntary contract with them. The contract would enable the enrolees to enjoy some additional entitlements (such as free health check-up) on top of the existing price advantage of CHCs over hospitals [6, 9]. However, patient perception on quality of care is usually the most important factor for consideration in their choice of care provider [10]. Indeed, quality of primary care services has attracted increasing attention as the number of primary care facilities increased. There exist serious quality challenges in the primary care sector in China, including a shortage of skilled workforce, fragmentation of medical and public health services, and deviations from good practice. Community-based primary care facilities are usually deemed inferior to their hospital counterparts in quality of patient care [11].

This study aimed to assess the preference of the existing CHC consumers to consider community-based primary care facilities as their first choice for medical attention. Consumers may visit CHCs for various purposes. There is a paucity in the literature documenting whether they would take community-based primary care facilities as a first choice for medical attention, despite extensive studies into the performance of primary care in China [12–14].

## Methods

A cross-sectional survey of CHC patients was conducted in Wuhan, China, in 2019 (prior to the outbreak of COVID-19). The study protocol was approved by the Ethics Committee of Tongji Medical College, Huazhong University of Science and Technology research ethics committee. Implied informed consent was obtained prior to the commencement of each survey.

### Study setting

The study was conducted in Wuhan, the capital of Hubei province in central China. Wuhan reported an average gross domestic product (GDP) of 145,545 Yuan (US$21,100) per capita in 2019, ranking on top 9 among the 35 provincial capital and central-governed cities in mainland China [15]. There are 133 CHCs across the 13 districts in Wuhan [16], servicing more than 11 million populations.

### Study participants

Study participants were recruited using a two-stage sampling strategy. At the first stage, 55 CHCs were randomly selected from the 13 districts of Wuhan: 1 to 8 in each district in proportion to their number of CHCs (stratified random sampling). At the second stage, 30 or more patients in each CHC who came to see a medical doctor and/or receive medical treatment at the time of data collection were approached (opportunity sampling) and invited to participate in this study. This resulted in a minimal sample size of 1650 to enable detection of significance for an odds ratio of 1.5 with 0.05 α error probability and 0.95 statistical power (1-β) [17].

### Data collection

Data were collected over the period from March to June in 2019. Five groups of trained investigators were recruited from the universities in Wuhan, each comprising at least two students led by a researcher. They approached the CHC visitors on the day of data collection, assessed their eligibility, explained the purpose of the study, and invited those who were eligible and willing to participate to complete the electronic questionnaire using their own mobile phone through QR scanning. On average, each survey took 5–10 minutes to complete. The survey was anonymous and voluntary. The data collection activities ended once the minimal sample size was reached in each participating CHC. In total, 1919 complete questionnaires were returned.

### Measurements

The self-administered questionnaire was developed by the research team in reference to the existing literature, which comprised three sections. Section one collected data in relation to the sociodemographic characteristics of the participants, such as gender, age (years), marital status (single, married, divorced, widowed), educational attainment (university vs non-university), employment (employed, retired, flexible, farming, unemployed, student), and personal monthly income (categorised into four groups: ≤2000, 2001–4000, 4001–6000, >6000 Yuan). Section two measured preferred first choice for medical attention using one single item: ‘When you feel unwell, which of the following medical institutions (CHCs and community health clinics, private clinics, hospitals) will you choose to visit first?’. Section three investigated the experience of the study participants in line with the ServQUAL framework [18–20]. Respondents were asked about how long it took to reach the nearest CHC and how satisfied they felt with the CHCs in terms of their facility and equipment, quality of professional services (medical, nursing, and pharmaceutical), interpersonal manner and responsiveness, time spent with the medical doctor, and accessibility and convenience of the treatment process. These aspects are common domains for measuring patient experience [21], which were adapted to the specific context of CHCs in China through expert consultations. They are also aligned with the quality of patient care framework developed by the European Regional Office of the World Health Organization [22]. Empirical evidence shows that patient decisions are shaped by their experience, including their confidence and trust in the care providers [23, 24].

In this study, eight items were developed to measure the above-mentioned aspects of patient experience (Questionnaire in English and Chinese). Each item was rated on a five-point Likert scale, ranging from 1 “very satisfied/strongly agree” to 5 “very dissatisfied/strongly disagree”.

### Statistical analysis

Data were entered into EpiData Info 3.1 (The EpiData Association, Denmark). Statistical analyses were performed using SPSS 19 (IBM).

The sociodemographic characteristics of respondents and their experience with CHCs were described through frequency distributions. The preferred first choice of care providers of the study participants was grouped into two categories: community-based primary care facilities and hospitals. Differences in the choice among the respondents with different characteristics and experiences were tested using Chi-square tests. Multivariate logistic regression models were established to determine the factors associated with the patient choice after adjustment for variations in other variables. We categorised predictors into two groups: sociodemographic characteristics of study participants and patient experience with CHCs. Three regression models were established accordingly: model one included sociodemographic characteristics only; model two included patient experience with CHCs only; model three included both groups of predictors. Collinearity of the independent variables were diagnosed using variance inflation factor (VIF) and tolerance. A VIF value of greater than 10 or 5 and/or tolerance below 0.2 or 0.1 usually indicate potential collinearity problems [25].

## Results

### Sociodemographic characteristics of respondents

More than two thirds of the study participants were women. Most were in the age between 26 and 45 years (58.1%) and had a university qualification (55.0%). The vast majority were married (75.0%) and employed (64.5%) at the time of the survey. More than 45% reported a personal monthly income between 2001 and 4000 Yuan, compared with 27% between 4001 and 6000 Yuan (Table 1).

**Table 1:** Sociodemographic characteristics of study participants who visited community health centres and their first choice for medical attention (n = 1919).

Characteristics	Sample size n (%)	First choice for medical attention				P*
		Community-based primary care facility		Hospital		
		N	%	N	%	
Gender						0.010
Male	592(30.8)	473	79.9	119	20.1	
Female	1327(69.2)	988	74.5	339	25.5	
Age (years)						<0.001
<26	314(16.4)	225	71.7	89	28.3	
26–35	662(34.5)	459	69.3	203	30.7	
36–45	452(23.6)	358	79.2	94	20.8	
46–55	269(14.0)	227	84.4	42	15.6	
>55	222(11.6)	192	86.5	30	13.5	
Marital status						0.132
Single	421(21.9)	304	72.2	117	27.8	
Married	1440(75.0)	1109	77.0	331	23.0	
Divorced	39(2.0)	32	82.1	7	17.9	
Widowed	19(1.0)	16	84.2	3	15.8	
University qualification						0.001
No	863(45.0)	689	79.8	174	20.2	
Yes	1056(55.0)	772	73.1	284	26.9	
Employment						0.002
Employed	1237(64.5)	944	76.3	293	23.7	
Retired	205(10.7)	173	84.4	32	15.6	
Flexible	246(12.8)	173	70.3	73	29.7	
Farming	33(1.7)	30	90.9	3	9.1	
Unemployed	61(3.2)	42	68.9	19	31.1	
Student	137(7.1)	99	72.3	38	27.7	
Average personal monthly income (Chinese Yuan)						0.021
≤2000	258(13.4)	194	75.2	64	24.8	
2001–4000	877(45.7)	677	77.2	200	22.8	
4001–6000	520(27.1)	408	78.5	112	21.5	
>6000	264(13.8)	182	68.9	82	31.1	
**Total**	1919(100)	1461	75.1	478	24.9	

* p values of Chi-square tests

### First choice for medical attention

About 75% of the respondents would choose community-based primary care facilities as a first choice for medical attention. Those who were male, younger (≤35 years), had a university qualification, did not have a stable job, and reported a higher personal income were less likely than others to identify community-based primary care facilities as a first choice for medical attention (Table 1).

### Patient experience in community health services

About 40% of the respondents had access to a CHC within a 10-minute walking distance, compared with 43% within 11 to 20 minutes. Only 5% needed to travel more than 30 minutes to reach a CHC. Geographic convenience was associated with the choice of community-based primary care facilities as a first provider for medical attention (p<0.001).

The study participants expressed a very high level of satisfaction towards CHCs, with over 90% being satisfied or very satisfied. The responses were largely consistent across those with different sociodemographic characteristics (S1 Table). Courtesy and responsiveness, time spent in communication, and nursing services were received relatively higher levels of satisfaction compared with pharmacy services, medical equipment, basic facility, medical services, and treatment process (Table 2).

**Table 2:** Patient experience in community health services and first choice for medical attention (n = 1919).

Patient satisfaction with community health services	n (%)	First choice for medical attention				P*
		Community-based primary care facility		Hospital		
		N	%	N	%	
**Basic facility**						<0.001
Very satisfied	1346(70.1)	1086	80.7	260	19.3	
Satisfied	430(22.4)	307	71.4	123	28.6	
Not satisfied	143(7.5)	68	47.6	75	52.4	
**Medical equipment**						<0.001
Very satisfied	1295(67.5)	1041	80.4	254	19.6	
Satisfied	475(24.8)	348	73.3	127	26.7	
Not satisfied	149(7.8)	72	48.3	77	51.7	
**Medical services**						<0.001
Very satisfied	1295(67.5)	1045	80.7	250	19.3	
Satisfied	511(26.6)	366	71.6	145	28.4	
Not satisfied	113(5.9)	50	44.2	63	55.8	
**Nursing services**						<0.001
Very satisfied	1392(72.5)	1115	80.1	277	19.9	
Satisfied	463(24.1)	321	69.3	142	30.7	
Not satisfied	64(3.3)	25	39.1	39	60.9	
**Treatment process**						<0.001
Very satisfied	1328(69.2)	1071	80.6	257	19.4	
Satisfied	496(25.8)	352	71.0	144	29.0	
Not satisfied	95(5.0)	38	40.0	57	60.0	
**Time spent with doctor**						<0.001
Very satisfied	1376(71.7)	1107	80.5	269	19.5	
Satisfied	459(23.9)	320	69.7	139	30.3	
Not satisfied	84(4.4)	34	40.5	50	59.5	
**Courtesy and responsiveness**						<0.001
Very satisfied	1452(75.7)	1170	80.6	282	19.4	
Satisfied	410(21.4)	270	65.9	140	34.1	
Not satisfied	57(3.0)	21	36.8	36	63.2	
**Pharmacy services**						<0.001
Very satisfied	1192(62.1)	972	81.5	220	18.5	
Satisfied	575(30.0)	418	72.7	157	27.3	
Not satisfied	152(7.9)	71	46.7	81	53.3	

Note: the category of “not satisfied” covers “slightly dissatisfied”, “dissatisfied” and “very dissatisfied”.

* p values of Chi-square tests.

Those who were satisfied with the eight aspects of CHCs were more likely (p<0.05) than those who were not satisfied to take community-based primary care facilities as a first choice for medical attention (Table 2).

Three logistic regression models were developed. No significant collinearity problems among the independent variables were identified. Of the sociodemographic characteristics, male gender, older age, and lower personal income were identified as significant predictors of having community-based primary care facilities as a first choice for medical attention (model one in Table 3). Geographic proximity and higher levels of satisfaction with the basic facility, courtesy and responsiveness, and pharmacy services in the CHCs were associated with a higher likelihood of taking community-based primary care facilities as a first choice for medical attention (model two in Table 3). The final model (model three) included all of the predictors in model one and model two, which increased the R^2^ significantly. Apart from gender, the significant predictors identified in model one and model two remained statistically significant (Table 3).

**Table 3:** Predictors of having community-based primary care facilities as a first choice for medical attention: Results of multivariate logistic regression model (n = 1919).

Predictor	Model One			Model Two			Model Three		
	AOR	95% Confidence Interval		AOR	95% Confidence Interval		AOR	95% Confidence Interval	
		Lower bound	Higher bound		Lower bound	Higher bound		Lower bound	Higher bound
**Sociodemographic characteristics**									
**Gender** (Ref. = Female)									
Male	**1.37** *	1.070	1.777				1.310	0.999	1.718
**Age** (Ref. =“>55” Years)									
<26	**0.348** **	0.166	0.730				**0.290** **	0.129	0.652
26–35	**0.334** **	0.167	0.665				**0.252** ***	0.118	0.539
36–45	0.536	0.265	1.083				0.434*	0.201	0.939
46–55	0.732	0.375	1.426				0.694	0.333	1.444
**University qualification** (Ref. = Yes)									
No	1.208	0.941	1.550				1.252	0.962	1.629
**Employment** (Ref. = Student)									
Employed	1.097	0.594	2.025				1.236	0.643	2.379
Retired	0.746	0.304	1.829				0.693	0.262	1.834
Flexible	0.785	0.409	1.505				0.799	0.401	1.594
Farming	2.123	0.569	7.924				2.872	0.712	11.588
Unemployed	0.719	0.334	1.549				0.710	0.313	1.609
**Average personal monthly income** (Ref. =“>6000” Chinese yuan)									
≤2000	1.553	0.934	2.583				**1.836** *	1.066	3.161
2001–4000	**1.492** *	1.071	2.079				**1.637** **	1.150	2.330
4001–6000	**1.560** *	1.103	2.206				**1.603** *	1.111	2.312
**Satisfaction with community health services**									
**Basic facility** (Ref. =“Not satisfied”)									
Very satisfied				**1.998** *	1.151	3.469	**2.068** *	1.176	3.637
Satisfied				1.452	0.868	2.430	1.441	0.853	2.435
**Medical equipment** (Ref. =“Not satisfied”)									
Very satisfied				0.973	0.504	1.882	1.221	0.620	2.402
Satisfied				1.370	0.798	2.354	1.680	0.966	2.921
**Medical services** (Ref. =“Not satisfied”)									
Very satisfied				1.408	0.642	3.085	1.501	0.664	3.394
Satisfied				1.355	0.711	2.583	1.534	0.789	2.981
**Nursing services** (Ref. =“Not satisfied”)									
Very satisfied				0.784	0.311	1.972	0.688	0.267	1.778
Satisfied				0.986	0.433	2.248	0.848	0.365	1.971
**Treatment process** (Ref. =“Not satisfied”)									
Very satisfied				1.318	0.615	2.828	1.407	0.643	3.076
Satisfied				1.317	0.680	2.551	1.399	0.713	2.746
**Time spent in communication** (Ref. =“Not satisfied”)									
Very satisfied				0.826	0.336	2.032	0.746	0.293	1.904
Satisfied				0.956	0.442	2.069	0.878	0.394	1.959
**Courteous and comprehensive response** (Ref. =“Not satisfied”)									
Very satisfied				**2.717** *	1.032	7.157	**2.800** *	1.030	7.610
Satisfied				1.418	0.601	3.348	1.403	0.578	3.408
**Pharmacy services** (Ref. =“Not satisfied”)									
Very satisfied				**2.222** **	1.349	3.659	**2.220** **	1.325	3.722
Satisfied				**1.701** *	1.081	2.678	**1.675** *	1.048	2.677
**Walking distance** (Ref. = Over 30 minutes)									
≤10				**3.592** ***	2.290	5.634	**3.848** ***	2.398	6.175
11–20				**3.236** ***	2.072	5.055	**3.417** ***	2.140	5.456
21–30				**2.023** **	1.220	3.355	**2.163** **	1.277	3.663
**R** ^**2**^	0.034			0.077			0.117		

Note: AOR–Adjusted Odds Ratio

*p<0.05

**p<0.01

***p<0.001

## Discussion

Although community-based primary care facilities are attracting more patients in China, not all of the patients take these facilities as a first point of contact for medical attention. This study shows that one quarter of the CHC patients would not consider community-based primary care facilities as their first choice for medical attention, despite an overwhelming expression (>90%) of satisfaction with CHCs. This raises a serious question about the capability of CHCs to achieve the full potential of their comprehensive functions assigned by the government. A community survey conducted in 2015 in one of China’s most developed regions Zhejiang showed that 55% residents visited CHCs for outpatient care at least once in the past year, compared with 64% visiting hospital-based clinics at least once in the past year [26]. It is reasonable to assume that the patients who do not visit CHCs are even less likely to consider community-based primary care facilities as a first choice for medical attention. Indeed, even in a system where the CHCs had received alliance support from the large hospitals, less than 65% of the patients were willing to undertake a first visit to primary care facilities [27].

It appears that the willingness of people to take community-based primary care facilities as a first choice for medical attention was growing over the past few years. In a 2013 survey of CHCs across six provinces, we found that only 62% of the patients preferred to let CHCs initiate medical care for them when they felt unwell although the sample size was relatively small [28]. This figure increased to 75% according to the findings of this study. The increase coincides with the improved capacity and quality of patient care delivered by CHCs as a result of increased governmental investment in primary care in the recent health system reforms [6]. However, we cannot exclude the possibility of CHCs losing some patients who felt disappointed with their CHC experience.

In China, consumers have the autonomy to choose between primary care institutions and hospitals as their first point of contact for medical care. The patient choice is determined by many factors. Some are predominantly driven by the price and convenience, while others are driven by the perceived quality of care depending on the complex needs of medical care [29]. This study revealed that older patients and those with a lower income are more likely to take community-based primary care facilities as a first choice for medical attention. These results are consistent with the findings of previous studies [30–32]. Obviously, financial concern has continued to play an important role in the patient choice of care providers despite near universal coverage of social health insurance programs in China [33].

Geographic proximity is the most powerful predictor of the patient choice according to the regression modelling established in this study. This is not surprising given that convenience and easy access is perhaps the most visible advantage of community-based primary care facilities over hospitals [6]. Previous studies showed that distance is a significant influencing factor on the patient choice of care providers [9, 34]. The Chinese government intends to make CHCs available to the entire populations within a 15-minute walking distance [6].

This study found that patient satisfaction with the basic facility of CHCs and the courtesy and responsiveness of the services makes a significant difference in the patient choice. However, patient satisfaction with the medical and nursing services does not predict the patient choice. This may have reflected the overall tendency in China to rely on hospitals to ensure the technical quality of medical care, including for primary care services. These results are consistent with some findings of previous studies. Good facility is a tangible sign for the safety and quality of care [12]. It may help increase the confidence and trust of consumers in primary care institutions [9, 35]. When the technical quality of health care services is not taken as a paramount consideration, courtesy and responsiveness may become even more important for the patient choice [36, 37].

Patient satisfaction with the pharmacy services in CHCs was found to be another significant predictor of the patient choice in this study. Pharmacy services have been a major policy focus of health reforms in China [38]. Lower-priced medicines are guaranteed through the zero-markup policy and the centralised procurement system, albeit being restricted to the essential medicines list [39]. The relatively low level of patient satisfaction on pharmacy services found in this study is perhaps a reflection of the low availability of medicines in CHCs due to distribution failures as revealed in previous studies [40–42]. In addition, there is a lack of qualified community pharmacist workforce in China [39]. Nevertheless, pharmacy services have remained to be a significant determinant of the patient choice of first providers for medical attention.

Serious challenges lie ahead in China’s health system reform. In many developed countries, primary care providers are highly trusted and are assigned with a “gatekeeping” role [43, 44]. In China, however, there is still a lack of consensus among the public regarding the “gatekeeping” role of primary care providers despite a significant growth and improvement in primary care services [14, 28]. The healthcare delivery system is still dominated by hospitals. Arguably, whether primary care facilities can play a “gatekeeping” role depends on the power balance between the primary care and the hospital sectors.

The findings of this study offer some lessons to the low- and middle-income countries (LMICs) in their efforts to achieve universal health coverage. Empirical evidence shows that primary care-dominated health systems perform better than those dominated by hospitals [45, 46]. However, in many LMICs, community-based primary care services are often featured with a lack of skilled workforce and poor quality of patient care [47]. When hospital services become more affordable, more patients may choose hospitals as their first choice for medical attention. This calls for increasing attention to formal medical education of primary care workers.

### Limitations

This study has several limitations. The study participants were restricted to those who sought medical care from CHCs, which can result in an overestimation of the patient agreement with community-based primary care facilities as a first care provider. The study sample was drawn from urban CHCs, which cannot reflect the situation of rural residents. Female patients were over presented in this study, partly because there were more female patients than male patients in CHCs and partly because female patients were more willing to cooperate in the study. The study adopted a cross-sectional design and no causal relationships should be assumed. Although China implemented a top-down health reform agenda, regional disparities in socioeconomic development can still have a significant impact on the primary care development, which may limit the generalisability of this study.

## Conclusion

Since 2009, the Chinese government has made great efforts to revitalise its primary care system through a series of policy initiatives. The rapid development of CHCs has provided consumers with more convenient access to medical care and public health services. The consumers of CHCs are generally satisfied with the services they received. However, one quarter of the CHC patients in this study are yet to be convinced to accept community-based primary care facilities as a preferred first provider for medical care, let alone the general public. Patient experience with CHCs, in particular in responsiveness and pharmacy services, is associated with the choice of community-based primary care facilities as a first provider for medical care. Currently, CHCs are likely to be seen as a key player in supplementing the hospital-based services, especially for those who are older and more price sensitive. There is a long way to go to achieve the governmental vision of developing a primary care-dominated health care system, in which common health problems are treated in primary care facilities while hospitals can focus on referred difficult cases.

## Supporting information

S1 Table Patient experience in community health services (n = 1919). (DOCX)

S1 Appendix Questionnaire in English and Chinese. (DOCX)

S1 Data Data for the survey. (XLS)
