# Supplementary material for: Patient experience in community health services and first choice for medical attention: A cross-sectional study in Wuhan, China
Source: PLoS One. 2023 Jul 25;18(7):e0288164. doi: 10.1371/journal.pone.0288164 (PMC10368283; doi:10.1371/journal.pone.0288164)
Supplement: S1 Table — (DOCX) [file pone.0288164.s001.docx]

**Supplementary Table S1. Patient experience in community health services (n=1919)**

|  | Satisfaction with basic facility | | Satisfaction with medical equipment | | Satisfaction with medical services | | Satisfaction with nursing services | | Satisfaction with treatment process | | Satisfaction with time spent with doctor | | Satisfaction with courtesy and responsiveness | | Satisfaction with pharmacy services | |
| --- | --- | --- | --- | --- | --- | --- | --- | --- | --- | --- | --- | --- | --- | --- | --- | --- |
|  | % | p | % | p | % | p | % | p | % | p | % | p | % | p | % | p |
| Gender |  | 0.127 |  | 0.198 |  | 0.977 |  | 0.370 |  | 0.227 |  | 0.482 |  | 0.865 |  | 0.046 |
| Male | 93.9 |  | 93.4 |  | 94.1 |  | 96.1 |  | 95.9 |  | 96.1 |  | 97.1 |  | 93.9 |  |
| Female | 91.9 |  | 91.7 |  | 94.1 |  | 96.9 |  | 94.6 |  | 95.4 |  | 97.0 |  | 91.3 |  |
| Age (Years) |  | 0.150 |  | 0.046 |  | 0.118 |  | 0.024 |  | 0.218 |  | 0.119 |  | 0.087 |  | 0.475 |
| <26 | 91.4 |  | 92.0 |  | 93.0 |  | 94.9 |  | 93.3 |  | 93.9 |  | 94.9 |  | 94.6 |  |
| 26–35 | 93.7 |  | 94.3 |  | 95.3 |  | 96.8 |  | 95.9 |  | 95.5 |  | 97.3 |  | 91.4 |  |
| 36–45 | 93.1 |  | 91.4 |  | 94.5 |  | 97.6 |  | 95.4 |  | 96.5 |  | 98.0 |  | 92.0 |  |
| 46–55 | 89.2 |  | 88.5 |  | 91.1 |  | 94.8 |  | 93.3 |  | 94.4 |  | 96.3 |  | 91.1 |  |
| >55 | 93.7 |  | 92.8 |  | 95.0 |  | 99.1 |  | 96.4 |  | 98.2 |  | 98.2 |  | 91.9 |  |
| Marital status |  | 0.497 |  | 0.127 |  | 0.097 |  | 0.091 |  | 0.250 |  | 0.097 |  |  |  | 0.399 |
| Single | 91.4 |  | 91.2 |  | 92.9 |  | 94.8 |  | 93.6 |  | 93.8 |  | 95.5 | 0.122 | 93.8 |  |
| Married | 92.8 |  | 92.6 |  | 94.6 |  | 97.2 |  | 95.5 |  | 96.0 |  | 97.4 |  | 91.5 |  |
| Divorced | 92.3 |  | 84.6 |  | 87.2 |  | 97.4 |  | 92.3 |  | 100 |  | 100 |  | 94.9 |  |
| Widowed | 100.0 |  | 100.0 |  | 100 |  | 100 |  | 100 |  | 100 |  | 100 |  | 89.5 |  |
| University qualification |  | 0.957 |  | 0.865 |  | 0.671 |  | 0.649 |  | 0.787 |  | 0.284 |  | 0.659 |  | 0.653 |
| No | 92.6 |  | 92.1 |  | 93.9 |  | 96.9 |  | 94.9 |  | 96.2 |  | 97.2 |  | 91.8 |  |
| Yes | 92.5 |  | 92.3 |  | 94.3 |  | 96.5 |  | 95.2 |  | 95.2 |  | 96.9 |  | 92.3 |  |
| Employment |  | 0.623 |  | 0.188 |  | 0.665 |  | 0.510 |  | 0.435 |  | 0.046 |  | 0.482 |  | 0.231 |
| Employed | 92.0 |  | 91.4 |  | 94.2 |  | 96.5 |  | 94.6 |  | 95.5 |  | 97.1 |  | 91.1 |  |
| Retired | 93.2 |  | 92.2 |  | 93.7 |  | 98.5 |  | 95.6 |  | 97.6 |  | 98.5 |  | 92.7 |  |
| Flexible | 94.3 |  | 95.5 |  | 94.3 |  | 96.3 |  | 96.7 |  | 96.7 |  | 97.2 |  | 94.7 |  |
| Farming | 90.9 |  | 87.9 |  | 93.9 |  | 97.0 |  | 90.9 |  | 87.9 |  | 93.9 |  | 93.9 |  |
| Unemployed | 96.7 |  | 96.7 |  | 98.4 |  | 98.4 |  | 98.4 |  | 98.4 |  | 95.1 |  | 90.2 |  |
| Student | 92.0 |  | 92.7 |  | 92.0 |  | 94.9 |  | 94.9 |  | 92.7 |  | 95.6 |  | 95.6 |  |
| Average personal monthly income (Chinese Yuan) |  | 0.111 |  | 0.091 |  | 0.027 |  | 0.032 |  | 0.141 |  | 0.080 |  | 0.001 |  | 0.227 |
| ≤2000 | 92.2 |  | 92.6 |  | 92.6 |  | 95.0 |  | 93.8 |  | 93.4 |  | 93.8 |  | 93.0 |  |
| 2001–4000 | 91.1 |  | 90.6 |  | 92.8 |  | 96.0 |  | 94.2 |  | 95.3 |  | 96.7 |  | 90.9 |  |
| 4001–6000 | 94.2 |  | 93.5 |  | 95.8 |  | 98.5 |  | 96.5 |  | 97.3 |  | 98.3 |  | 93.8 |  |
| >6000 | 94.3 |  | 94.7 |  | 96.6 |  | 97.0 |  | 96.2 |  | 95.5 |  | 98.9 |  | 91.7 |  |
| Total | 92.5 |  | 92.2 |  | 94.1 |  | 96.7 |  | 95.0 |  | 95.6 |  | 97.0 |  | 92.1 |  |
