# Supplementary material for: Patient experience in community health services and first choice for medical attention: A cross-sectional study in Wuhan, China
Source: PLoS One. 2023 Jul 25;18(7):e0288164. doi: 10.1371/journal.pone.0288164 (PMC10368283; doi:10.1371/journal.pone.0288164)
Supplement: S1 Appendix — (DOCX) [file pone.0288164.s002.docx]

**Survey on patient experience in primary health care institutions**

**Section One: Characteristics of Study Participants**

1. Gender

(1) Male

(2) Female

2. Age: (Years)

3. Marital status

(1) Single

(2) Married

(3) Divorced

(4) Widowed

4. Educational attainment

(1) Primary school or below

(2) Secondary school (including vocational training)

(3) University degree

5. Employment

(1) Employed

(2) Retired

(3) Flexible

(4) Farming

(5) Unemployed

(6) Student

6. Average individual monthly income (Chinese Yuan)

(1) ≤2000

(2) 2001–4000

(3) 4001–6000

(4) >6000

**Section Two: First Choice for Medical Attention**

7. When you feel unwell, which of the following medical institutions will you choose to visit first?

(1) Community health services (including community health centres, township health centres, and their outreach stations)

(2) Hospitals

(3) Private clinics

**Section Three: Patient Experience in Community Health Services**

8. The basic facilities (seats, water dispensers, signage, air conditioning, elevators, etc.) are good

(1) Strongly agree (2) Agree (3) Slightly disagree (4) Disagree (5) Strongly disagree

9. The diagnostic and treatment devices can meet the essential needs of patients

(1) Strongly agree (2) Agree (3) Slightly disagree (4) Disagree (5) Strongly disagree

10. The medical doctors provide good diagnostic and treatment services

(1) Strongly agree (2) Agree (3) Slightly disagree (4) Disagree (5) Strongly disagree

11. The nurses provide highly-skilled nursing care

(1) Strongly agree (2) Agree (3) Slightly disagree (4) Disagree (5) Strongly disagree

12. The treatment process is convenient and easy to access

(1) Strongly agree (2) Agree (3) Slightly disagree (4) Disagree (5) Strongly disagree

13. The frequency and time spent with my doctor is adequate

(1) Strongly agree (2) Agree (3) Slightly disagree (4) Disagree (5) Strongly disagree

14. The health workers are patient and responsive

(1) Strongly agree (2) Agree (3) Slightly disagree (4) Disagree (5) Strongly disagree

15. Are you satisfied with the pharmacy services in the facility?

(1) Very satisfied (2) Satisfied (3) Not so much (4) Dissatisfied (5) Very dissatisfied

16. How long does it take you to reach the nearest community health centre?

(1) ≤10 minutes (2) 11–20 minutes (3) 21-30minutes (4) >30minutes

**基层医疗机构患者就医体验调查表**

**一、个人基本情况**

1.性别

（1）男

（2）女

2.年龄： （岁）

3.婚姻状况

（1）未婚

（2）已婚

（3）离异

（4）丧偶

4.文化程度

（1）小学及以下

（2）初中/高中（包括中专、技校）

（3）本科及以上

5.职业

（1）职工

（2）退休

（3）灵活就业

（4）农民

（5）无业

（6）学生

6.个人月均收入

（1）2000元及以下

（2）2001元-4000元

（3）4001元-6000元

（4）6000元以上

**二、首选就医机构**

7.您身体不舒服时，一般情况下会首先选择以下哪类医疗机构去看病？（单选）

（1）基层医疗机构（包括村卫生室、社区卫生服务中心、乡镇卫生院）

（2）大医院

（3）私人诊所

**三、患者就医体验**

8.基本设施完善（座椅、饮水机、指示牌、空调、电梯等）

（1）非常赞同 （2）赞同 （3）一般 （4）不赞同 （5）非常不赞同

9.诊疗设备齐全，基本满足患者的就医需求

（1）非常赞同 （2）赞同 （3）一般 （4）不赞同 （5）非常不赞同

10.医生诊疗技术水平较好

（1）非常赞同 （2）赞同 （3）一般 （4）不赞同 （5）非常不赞同

11.护士护理技能熟练

（1）非常赞同 （2）赞同 （3）一般 （4）不赞同 （5）非常不赞同

12.就诊流程便捷

（1）非常赞同 （2）赞同 （3）一般 （4）不赞同 （5）非常不赞同

13.医务人员与患者交流频次、时间充足

（1）非常赞同 （2）赞同 （3）一般 （4）不赞同 （5）非常不赞同

14.医护人员对患者咨询解答耐心

（1）非常赞同 （2）赞同 （3）一般 （4）不赞同 （5）非常不赞同

15.您对目前您在社区卫生服务机构内接受到的药学服务满意吗？

（1）非常满意 （2）满意 （3）一般 （4）不满意 （5）非常不满意

16.要到达距离您家最近的社区卫生服务中心，您一般花费多少时间？______

（1）10分钟及以下 （2）11分钟-20分钟 （3）21分钟-30分钟 （4）30分钟以上
